# Investigating the contributors to hit-and-run crashes using gradient boosting decision trees

**DOI:** 10.1371/journal.pone.0314939

**Published:** 2025-01-03

**Authors:** Baorui Han, Haibo Huang, Gen Li, Chenming Jiang, Zhen Yang, Zhenjun Zhu

**Affiliations:** 1 School of Automobile and Traffic Engineering, Nanjing Forestry University, Nanjing, Jiangsu, China; 2 China Institute of FTZ Supply Chain, Shanghai Maritime University, Shanghai, Shanghai, China; Tsinghua University, CHINA

## Abstract

A classification prediction model is established based on a nonlinear method—Gradient Boosting Decision Tree (GBDT) to investigate the factors contributing to a perpetrator’s escape behavior in hit-and-run crashes. Given the U.S. Crash Report Sampling System (CRSS) dataset, the model is trained and compared with the state-of-art methods (Classification and Regression Tree, Random Forest, and Logistic Regression). The results show that the GBDT outperforms other methods, achieving the lowest negative log-likelihood (0.282), misclassification rate (0.096), and the highest AUC (0.803). GBDT also demonstrates superior computational efficiency, with a LIFT value of 4.087, making it a more accurate and efficient model for predicting hit-and-run crashes compared to CART, Random Forest, and Logistic Regression. The results obtained from the GBDT show that the relative importance of *crash type* and *relation to trafficway* rank 4th and 5th, respectively. Neither is mentioned in previous studies, indicating that GBDT has the ability to mine hidden information. In addition, the interaction between influencing variables can also be obtained to investigate the joint effect of various variables. The results of this study have practical applications in hit-and-run incident prevention, accident safety analysis, and other engineering applications.

## Introduction

According to the World Health Organization’s report released in December 2023, approximately 1.19 million people die from road traffic accidents yearly [1]. Among different types of traffic accidents, hit-and-run crashes are one of the most harmful types, posing risks to the life safety of victims. Besides, hit-and-run crashes also inflict significant psychological pressure on the families of victims and exert a negative influence on the overall societal order and moral values. Therefore, hit-and-run is commonly defined as a crime according to the Road Traffic Safety Law worldwide, and it is strictly prohibited and severely punished by law [2]. However, hit-and-run is a covert behavior influenced by multiple factors, with interactive effects between these factors, making it exceptionally challenging to identify key factors. AS a result, predicting the occurrence of hit-and-run becomes highly difficult and complex.

Hit-and-run traffic accidents have become a serious societal issue, causing significant harm and impact on individuals, families, and society as a whole. Of the 7,388 pedestrian fatalities from traffic accidents that occurred in the United States in 2021 (NHTSA, 2022), 1,802 involved hit-and-run crashes, accounting for a percentage24% of the total. This represents the highest number of participants in hit-and-run crashes since 2005, as well as the largest annual proportion of participants in hit-and-run crashes in the history of the Fatal Analysis Reporting System. Behind each number lies a tragic loss of life and a family left abandoned (NHTSA, 2022). Thus, it is essential to investigate the contributing factors of hit-and-run accidents and propose targeted prevention and control measures.

Some studies have tried to investigate the key factors influencing the occurrence of hit-and-run crashes based on statistical analysis and modeling methods [3–5]. These methods can describe the impact level of different factors well but produce dissatisfied accuracy. Thus, machine learning methods have been introduced in recent years [6–8]. Despite the existing applications of machine learning in traffic safety, there remains a clear gap in applying interpretable machine learning models to understand the underlying factors influencing hit-and-run behavior. This study is the first to use GBDT to analyze hit-and-run crashes, utilizing the 2021 Crash Report Sampling System data from the National Highway Traffic Safety Administration (NHTSA) to identify key factors. Unlike black-box machine learning models, GBDT not only provides superior predictive performance but also enables the identification of variable importance and interactions. Its ability to mine hidden information from high-dimensional traffic data offers interpretable insights that can support policy recommendations aimed at preventing hit-and-run incidents.

The structure of this paper unfolds as follows. The Literature review section discusses empirical literature and summarizes the methodologies used in studying hit-and-run behavior. The Materials and Methods section describes the methods, data, and variables employed. The Results section presents the empirical results. The Discussion section provides a discussion of the results. Finally, the Conclusions section concludes the study, highlighting its limitations and avenues for future research.

## Literature review

Analysis of hit-and-run factors has received attention from researchers in recent decades. Early studies primarily focused on understanding the causal relationships between influencing factors and crash occurrences through statistical models. Researchers identified five key categories of factors: crash accident attributes, human attributes, vehicle attributes, road attributes, and environmental attributes. For instance, Tay et al. [3, 9] found that the object collided with, crash angle and several colliding vehicles significantly influence hit-and-run behavior by establishing binary logistic models. In terms of human attributes, Zhou et al. [10], Sivasankaran et al. [11], and Roshandeh et al. [12] highlighted the importance of human factors, such as improper driving behavior, driver distraction, and pedestrian safety, in predicting hit-and-run incidents. Macleod et al. [13] found that both the age of the driver and the age of the victim significantly impact drivers’ propensity to flee. In the aspect of vehicle attributes, Zhang et al. [14, 15] discovered that vehicle type and vehicle usage also have a certain impact on drivers’ behavior. Concerning road attributes, Aidoo et al. [16] analyzed the effects of road environmental conditions such as road linearity, road surface conditions, and central dividers on hit-and-run behavior. Das et al. [17] also unearthed the correlation between road geometric features and hit-and-run behavior. However, Fujita et al. [18] argued that the influence of road-related factors on reducing the likelihood of drivers fleeing after hitting pedestrians is limited. Most studies include environmental attributes due to the specificity of environmental factors. For instance, Jiang et al. [19] found that lighting conditions significantly affect drivers’ hit-and-run behavior in the study of hit-and-run accidents on urban overpass tunnels. Zhu et al. [20, 21] found that the impact of non-peak hours on hit-and-run behavior is more significant than during peak hours in the study of bicycle-car crash accidents. Lopez et al. [22] found that weekend factors are more significant in factors related to hit-and-run crashes after vehicle-bicycle crash accidents.

To model these factors, researchers have employed both statistical and machine learning approaches. Traditional statistical models have been widely used to identify significant predictors of hit-and-run behavior. For instance, Tay et al. [4], Macleod et al. [13], Aidoo et al. [16], Roshandeh et al. [12], Jiang et al. [23], established binary logistic regression models to identify significant influencing factors of hit-and-run behavior and predict the likelihood of hit-and-run occurrences. Jiang et al. [5, 19] employed ordered logit models to analyze the key factors influencing hit-and-run behavior. Liu et al. [24] later developed geographically weighted regression models for similar purposes. Despite the significant contributions of these models in identifying key influencing factors of hit-and-run behavior, they often require explicit expressions and frequently assume independence among variables, leading to limitations in capturing complex nonlinear effects of influencing factors and handling interactions among high-dimensional feature variables.

In contrast, machine learning models, which do not require such assumptions, have emerged as more powerful tools for identifying and predicting hit-and-run behavior. Models such as Classification and Regression Trees [25, 26], market basket analysis [17], and Fast and Frugal Trees [27] have been utilized to handle complex and high-dimensional datasets. Furthermore, integrated frameworks combining machine learning techniques with technologies like blockchain and edge computing have been proposed [28–30], enhancing the capacity for real-time analysis of hit-and-run data. Notably, Jha et al. [7, 31, 32] conducted comparative studies between almost all machine learning models and logistic regression models involving key influencing factors of hit-and-run behavior, indicating the superior performance of machine learning models in identifying and predicting hit-and-run crashes. Both Jha et al. [31] and Zhou et al. [25] demonstrated the excellent performance of Classification and Regression Trees in predicting hit-and-run behaviors in their studies, including prediction accuracy, classification performance, and the ability to process high-dimensional data. Studies have consistently demonstrated that machine learning models outperform traditional statistical methods in terms of prediction accuracy and the ability to process large-scale, high-dimensional data.

However, while machine learning models offer improved accuracy, they often function as "black box" systems, making it difficult to interpret how individual factors contribute to prediction results. This lack of transparency limits their usefulness in understanding the mechanisms driving hit-and-run behavior, an aspect that is as crucial as prediction accuracy in traffic safety research.

In summary, while traditional statistical models have been instrumental in identifying key influencing factors, their limitations in handling complex interactions have prompted a shift towards machine learning approaches. Although machine learning models show superior predictive performance, the challenge remains to develop interpretable models that can provide both accuracy and insights into the factors influencing hit-and-run behavior.

## Materials and methods

### Establishment of gradient boosting decision tree

**Fig 1:**
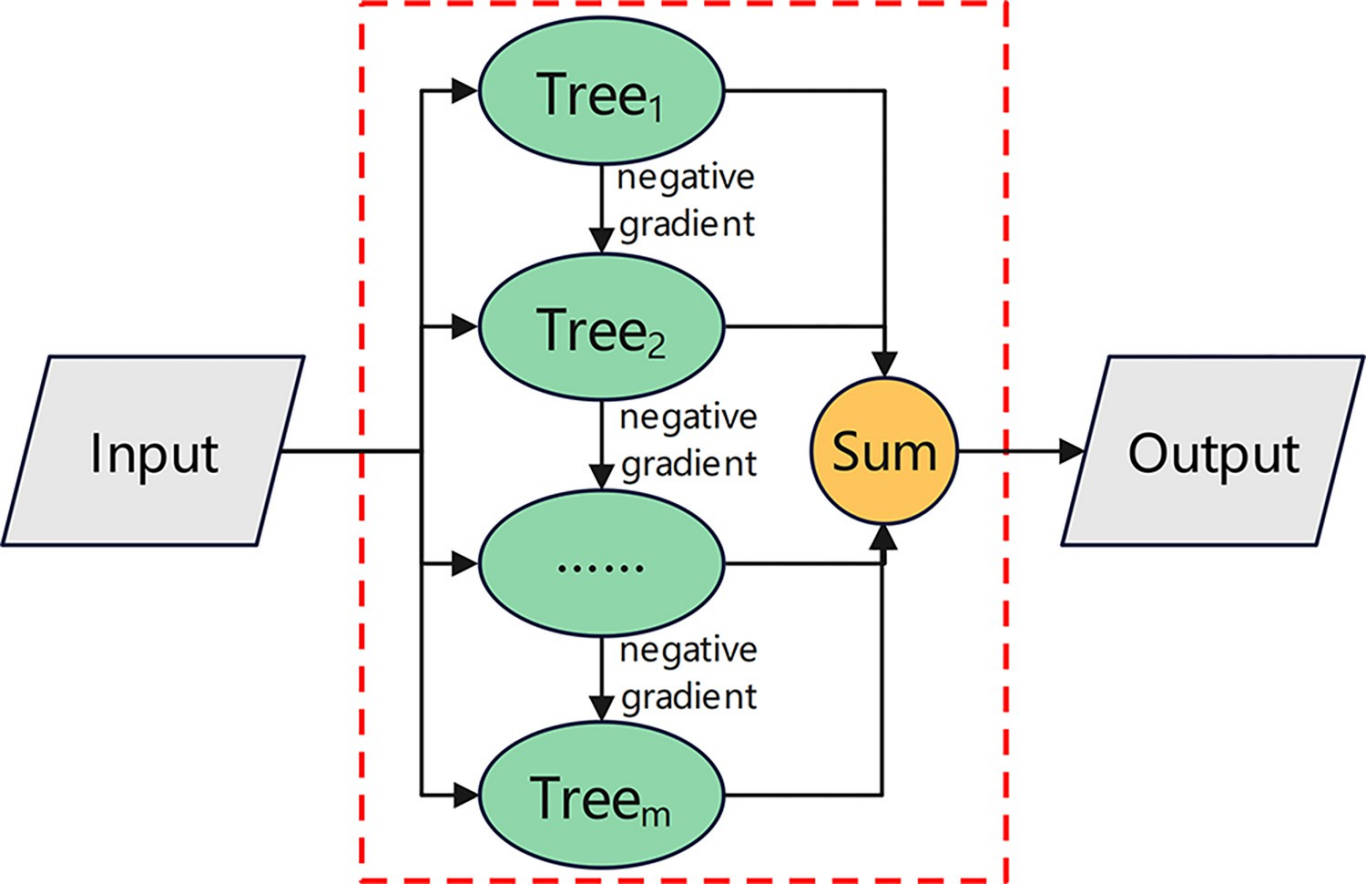
Basic principles of GBDT.

Gradient Boosting Decision Tree (GBDT) is a newly developed ensemble learning technique that uses Classification and Regressing Tree (CART) as its base learner and boosting technique to improve training accuracy iteratively. GBDT retains the interpretability of CART while addressing its susceptibility to small fluctuations in data. Proposed by Friedman [33], the core idea of GBDT is based on gradient boosting. GBDT minimizes the loss function, iteratively reduces residuals along the negative gradient direction of the loss function, and builds weak decision trees in this direction. Finally, the conclusions of all trees are accumulated to obtain the final prediction result, as shown in Fig 1.

Let *y* represents the driver’s hit-and-run situation, *x* represents the variables affecting the driver’s hit-and-run choice, and *N* represents the number of training samples. The process of building GBDT is as follows:

① Initialize the learner:

$$f_{0}(x)=\arg\;\min_{c}\sum_{i=1}^{N}L(y_{i},c)$$ (1)

where *c* is an approximate constant value that minimizes the loss function, and *L*(*y*_*i*_, *c*) is the loss function. In regression problems, mean square error loss is used:

$$L(y,f(x))={\left(y-f(x)\right)}^{2}$$ (2)

② Iteratively reduce residuals along the negative gradient direction:

When calculating the negative gradient for iteration *m* = 1,2,…,*M* concerning variable *i* = 1,2,…,*N*:

$$r_{mi}=-{\left[\frac{\partial L(y,f(x_{i}))}{\partial f(x_{i})}\right]}_{f(x)=f_{m-1}(x)}=y-f(x)$$ (3)

Fitting a regression tree for *r*_*mi*_, obtaining the leaf node regions *R*_*mj*_, *j* = 1,2,…,*J* for *m* trees. Calculate the best residual fitting values for each leaf node:

$$c_{mj}=\arg\;\min_{c}\sum_{x_{i}\in R_{mj}}L(y_{i},f_{m-1}(x_{i})+c)$$ (4)

③ Update the learner:

$$f_{m}(x)=f_{m-1}(x)+\sum_{j=1}^{J}c_{mj}I,x\in R_{mj}$$ (5)

where

$$I=\{\begin{array}{cc} 1, & x\in R_{mj}\\ 0, & x\notin R_{mj} \end{array}$$ (6)

④ Iterate M rounds to obtain the final hit-and-run model:

$$f(x)=f_{M}(x)=c+\sum_{m=1}^{M}\sum_{j=1}^{J}c_{mj}I,x\in R_{mj}$$ (7)

⑤ Calculate variable importance:

Based on the number of times a variable is selected as the splitting variable for regression tree *T*_*m*_, *m* = 1,2,…,*M* during the iteration *k*, calculate the importance of each variable:

$$I_{k}^{2}=\frac{1}{M}\sum_{m=1}^{M}I_{k}^{2}(T_{m})$$ (8)

### Data description and processing

This paper uses the 2021 traffic crash data from the NHTSA CRSS as a sample for model training and validation. The CRSS data collects crash report data provided by police departments from all 50 states in the United States. It details various factors of each traffic crash, including crash information, driver information, vehicle information, road information, and environmental information.

The crash accident data provided by CRSS include crash-related details such as the location, time, cause, type of crash, driver’s age, gender, attention level, injury status, risky driving behavior, vehicle type, usage, damage, and hit-and-run situations. However, due to the separate recording of the dataset and the presence of systematic errors and redundant information, the CRSS 2021 data undergo the following merging and filtering processes:

1. Match and merge separately recorded data based on the unique case number "CASENUM" in the dataset.
2. Records with missing values in critical variables (e.g., whether the crash involved a hit-and-run) were removed to avoid bias in the analysis. For non-critical variables, missing values were imputed using the mean or mode depending on the variable type. For continuous variables, such as speed limits, we used mean imputation. For categorical variables (e.g., weather, road surface conditions), mode imputation was applied.
3. Noise in the dataset arises from both human error in crash reporting and random fluctuations in recorded variables. We used z-scores to detect and remove extreme outliers in numerical variables (e.g., speed limits, crash angle). Data points with a z-score beyond ±3 standard deviations were considered outliers and were excluded from the analysis. To handle noisy fluctuations in continuous variables (e.g., speed limits), we applied a symmetrical exponential moving average (EMA) filter.

After processing, the CRSS 2021 data include a total of 54,187 crash accidents, among which there are 5,944 hit-and-run accidents, accounting for 10.97% of crash accidents. The hit-and-run and non-hit-and-run categories face a serious class imbalance issue, and data balancing processing is applied to the target variable during parameter calibration. Hit-and-run crashes constitute a relatively small proportion of total crashes in the dataset, leading to class imbalance in the binary classification target. To address this issue, we utilized the resampling techniques available in the data mining software. Specifically, random undersampling was applied to the majority class (non-hit-and-run crashes), while Synthetic Minority Over-sampling Technique (SMOTE) was used for the minority class. This ensured balanced class distribution in the training set, improving model performance and preventing the classifier from being biased toward the majority class.

### Data extraction and analysis

Based on previous research results [27, 28, 31, 32], the factors influencing driver hit-and-run include crash time (month *M*, hour *H*, day of week *WD*), lighting conditions *L*, weather conditions *WT*, road surface condition *VC*, road speed limit condition *VL*, driver’s age *A*, gender *S*, injury severity *I*, driver drinking in vehicle *VA*, Cargo Body Type *C*, and Special Use *SU*. However, driver hit-and-run is a covert behavior related to the exposure of drivers and the environment. Factors influencing driver exposure conditions, such as *Crash Type*, and factors influencing environmental exposure conditions, such as *Relation to Trafficway*, should be considered [34]. Therefore, these two factors are introduced into the model. Spearman correlation is initially used to analyze the correlation between hit-and-run accidents and various variables, as shown in Table 1. The data indicates weak correlations between hit-and-run accidents and various variables, suggesting that hit-and-run itself is a low-probability event, and studying hit-and-run problems is complex, with potential interactions among variables. Expressing this relationship with a linear model may not be effective, so consideration is given to a nonlinear model to address hit-and-run issues.

**Table 1:** Correlation coefficients between hit-and-run and various variables.

Explanatory Variable	*M*	*WD*	*H*	*RR*	*L*	*WT*	*SU*
Correlation Coefficient	-0.011	0.038	0.102	-0.153	-0.007	-0.017	0.261
P-value	0.013	0.000	0.000	0.000	0.114	0.000	0.000
*VA*	*VL*	*VC*	*AT*	*A*	*S*	*C*	*I*
0.016	-0.062	-0.011	-0.021	-0.077	0.031	0.259	-0.067
0.000	0.000	0.010	0.000	0.000	0.000	0.000	0.000

The variables influencing hit-and-run accidents were mainly selected from five aspects: accident characteristics, driver characteristics, vehicle characteristics, road features, and environmental features. A total of 16 research variables were chosen for analysis. Considering classification methods employed in previous studies, these 16 variables were categorized. The target variable, representing the driver’s hit-and-run status, is denoted as *HR*, as shown in Eq (9). The classification results for the remaining variables are provided in Table 2. The description of hit-and-run data for each variable is shown in Table 3.


$$HR=\{\begin{array}{cc} 1 & if\;hit\;and\;run\;occurs\\ 0 & otherwise \end{array}$$ (9)

**Table 2:** Classification of research variables.

Feature	Variable	Abbreviation	Variable Description
Accident Characteristics	*Day of Week*	*WD*	Weekday: *WD* = 0, Weekend: *WD* = 1
	*Hour*	*H*	6 a.m.–17 p.m.: *H* = 0, 18 p.m.–5 a.m.: *H* = 1
	*Month*	*M*	March-May: *M* = 1, June-August: *M* = 2, September-November: *M* = 3, December-February: *M* = 4
	*Crash Type*	*AT*	Rollover: *AT* = 0, Rear-end: *AT* = 1, Frontal: *AT* = 2, Lateral: *AT* = 3
Driver Characteristics	*Age*	*A*	Under 15 years old: *A* = 0, 16 years old and above: *A* = 1
	*Sex*	*S*	Male: *S* = 0, Female: *S* = 1
	*Injury Severity*	*I*	No injury: *I* = 0, Minor injury: *I* = 1, Light injury: *I* = 2, Severe injury: *I* = 3, Fatal injury: *I* = 4
	*Driver Drinking in Vehicle*	*VA*	No alcohol involved: *VA* = 0, Alcohol involved: *VA* = 1
Vehicle Characteristics	*Cargo Body Type*	*C*	Passenger vehicle: *C* = 0, Truck: *C* = 1
	*Special Use*	*SU*	Private vehicle: *SU* = 0, Public/commercial vehicle: *SU* = 1
Road Characteristics	*Speed Limit*	*VL*	No restriction: *VL* = 0, 5–30 km/h: *VL* = 1, 31–50 km/h: *VL* = 2, 51–120 km/h: *VL* = 3
	*Roadway Surface Condition*	*VC*	Wet/slippery lane: *VC* = 0, Dry lane: *VC* = 1
	*Relation to Trafficway*	*RR*	Outside roadway: RR = 0, On roadway: RR = 1
Environmental Characteristics	*Weather conditions*	*WT*	Sunny: *WT* = 0, Rainy/Snow: *WT* = 1
	*Light Condition*	*L*	Lighted: *L* = 0, Not Lighted: *L* = 1

**Table 3:** Description of hit-and-run data for each variable.

Feature	Variable	Value Range	Value	No. of crashes	No. of HR = 1	HR (%)
Accident Characteristics	*WD*	0/1	0	40458	4159	10.28
			1	13729	1785	13.00
	*H*	0/1	0	35976	3133	8.71
			1	18211	2811	15.44
	*M*	1–4	1	11485	1333	11.61
			2	13422	1512	11.27
			3	17739	1836	10.35
			4	11541	1263	10.94
	*AT*	0–3	0	8146	728	8.94
			1	12294	2077	16.89
			2	24144	1895	7.85
			3	9603	1244	12.95
Driver Characteristics	*A*	0/1	0	856	256	29.91
			1	53331	5688	10.67
	*S*	0/1	0	20810	2024	9.73
			1	33377	3920	11.74
	*I*	0–4	0	34667	4305	12.42
			1	7578	741	9.78
			2	6919	614	8.87
			3	4018	226	5.62
			4	1005	58	5.77
	*VA*	0/1	0	51033	5536	10.85
			1	3154	408	12.94
Vehicle Characteristics	*C*	0/1	0	49968	4306	8.62
			1	4219	1638	38.82
	*SU*	0/1	0	52678	5050	9.59
			1	1509	894	59.24
Road Characteristics	*VL*	0–3	0	10164	1277	12.56
			1	9144	1389	15.19
			2	23763	2350	9.89
			3	11116	928	8.35
	*VC*	0/1	0	8531	818	9.59
			1	44846	5126	11.43
	*RR*	0/1	0	2156	744	34.51
			1	52031	5200	9.99
Environmental Characteristics	*WT*	0/1	0	48580	5417	11.15
			1	5607	527	9.40
	*L*	0/1	0	8328	955	11.47
			1	45859	4989	10.88

## Results

### Model parameter calibration

The GBDT model is determined by six parameters: learning rate (*R*), maximum leaf nodes of a single decision tree (*J*), number of decision trees (*M*), attribute sampling count (*S*_*a*_), subsample ratio (*S*_*f*_), and test set ratio (Rt). In this paper, based on the processed CRSS 2021 data, the data mining software is utilized to handle class imbalance in the data and build the GBDT model. The positive target variable class ratio coefficient *P* is set to 0.11 according to the proportion of hit-and-run accidents in crash accidents. The learning rate *R* is set to Auto mode, ensuring the best fitting effect and the final value is obtained as 0.1 based on the model’s sample quantity and previous impact analysis studies. To achieve a balance between model performance and generalization ability, after repeated tuning, *J*, *S*_*a*_, and *S*_*f*_ are set to 6, 8, and 0.6, respectively. To prevent model overfitting, *R*_*t*_ is set to 0.4. The data mining software can automatically determine the optimal number of decision trees based on the objective function, and the final model is calibrated with *M* set to 338. The final parameter results of the GBDT model are shown in Table 4.

**Table 4:** GBDT model parameter calibration.

Model Parameter	Calibrated Value
Learning Rate (*R*)	0.1
Maximum Leaf Nodes of a Single Decision Tree (*J*)	6
Number of Decision Trees (*M*)	338
Attribute Sampling Count (*S*_*a*_)	8
Subsample Ratio (*S*_*f*_)	0.6
Test Set Ratio (*R*_*t*_)	0.4

All predictions in the GBDT modeling process with the data mining software are based on Eq (10). The GBDT model can provide Partial Dependency Plots (PDPs) for individual variables. Based on the partial dependency plot, the *O*_*i*_, *i* = 1,2,…,*n* value for the result of variable *x* can be obtained, where u is the number of categories for a variable result.

$$O_{i}=\frac{1}{2}\log(Odds)=\frac{1}{2}\log(\frac{p_{i}}{1-p_{i}})$$ (10)

where, *p*_*i*_ is the probability of hit-and-run when a certain result of the variable occurs.

Since the target variable HR in the study represents whether the driver hit-and-run, the model built is a binary model. For binary classification problems, such as predicting whether a driver will commit a hit-and-run, the odds ratio (OR) can be used to quantify the impact of a specific factor *x* on the target variable *y*. The odds ratio is defined in Eq (11) as:

$$OR=\frac{P(y=1|x)}{P(y=0|x)}$$ (11)

where *P*(*y* = 1|*x*) is the probability of a hit-and-run occurring given the value of *x*, and *P*(*y* = 0|*x*) is the probability of no hit-and-run. The odds ratio represents the change in odds when a certain factor is present.

### Model results analysis

**Fig 2:**
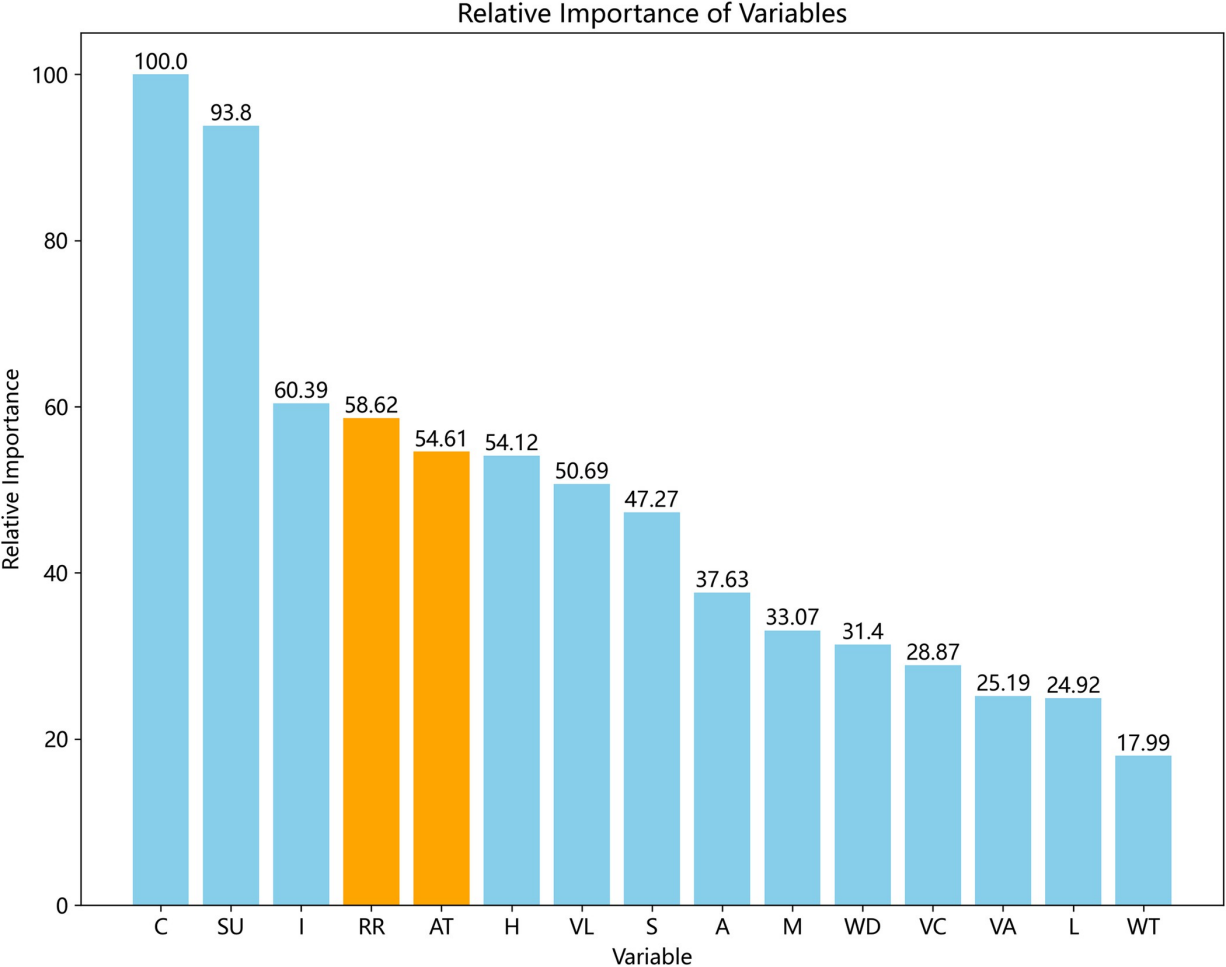
Relative importance of hit-and-run variables.

The GBDT calculates the importance of variables influencing hit-and-run accidents based on the number of times they are selected as splitting variables during the iteration. The data mining software automatically determines the relative importance of variables in the GBDT model based on the improvement in accuracy during the splitting process, as shown in Fig 2. The top three factors influencing crash hit-and-run crashes are identified as vehicle type, vehicle use, and severity of driver injuries. The relative importance rankings of *Relation to Trafficway* (*RR*) and *Crash Type* (*AT*) are 4th and 5th, respectively, aligning with the earlier conclusion that "*RR* and *AT* have an impact on hit-and-run accidents."

**Fig 3:**
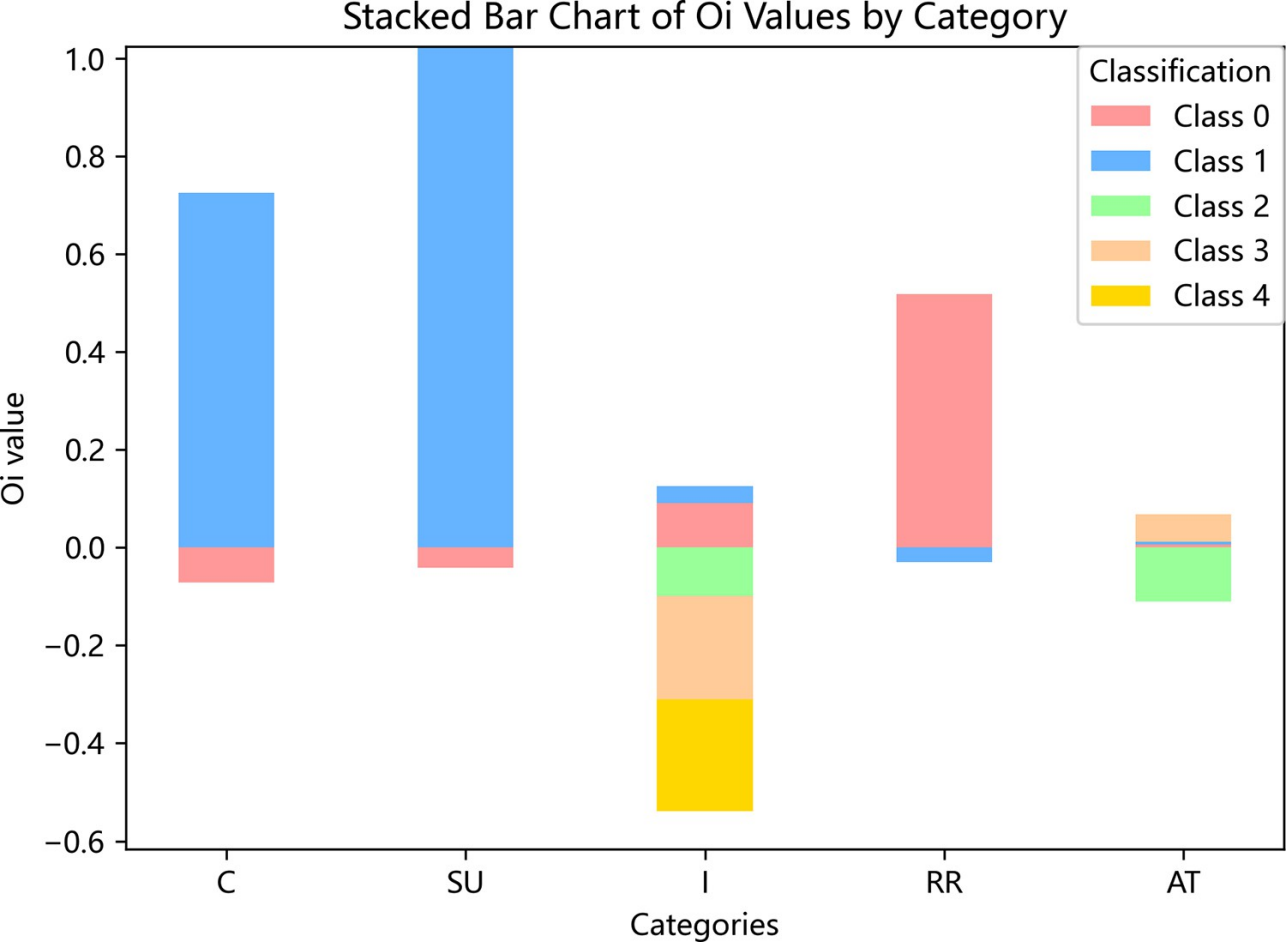
Output values (*O*_*i*_) of top five variables in importance ranking.

Fig 3 illustrates the impact of the top five factors influencing hit-and-run accidents, highlighting the effect of different categories within each factor. Notably, trucks have a greater positive influence on hit-and-run incidents compared to passenger vehicles. Public or commercial vehicles are more positively associated with hit-and-run accidents than private vehicles. Drivers who are injured have a greater negative influence on the likelihood of hit-and-run compared to uninjured drivers. Accidents occurring off the roadway are more likely to result in hit-and-run incidents than those on the roadway. Additionally, rear-end and lateral crashes have a higher probability of leading to hit-and-run crashes compared to front-end crashes.

The odds ratio (OR) is used to quantify the impact of variable *x* on the target variable, as shown in Table 5. For example, when the crash occurs on roadways (RR = 1) compared to outside roadway (RR = 0), the odds ratio is OR = 0.073, indicating a significantly lower likelihood of hit-and-run crashes when accidents occur on roadways. Similarly, when the crash type is lateral crashes (AT = 3) compared to rollover incidents (AT = 0), the odds ratio is OR = 1.225, suggesting a higher likelihood of hit-and-run behavior in side-impact crashes compared to single-vehicle incidents.

**Table 5:** Odds ratios of variables.

Relative variable	Variable value	OR
Truck: *C* = 1	Passenger vehicle: *C* = 0	0.022
Public/commercial vehicle: *SU* = 1	Private vehicle: *SU* = 0	0.004
No injury: *I* = 0	Minor injury: *I* = 1	0.951
Outside roadway: *RR* = 0	On roadway: *RR* = 1	0.073
Rollover: *AT* = 0	Lateral: *AT* = 3	1.225
6 a.m.–17 p.m.: *H* = 0	18 p.m.–5 a.m.: *H* = 1	3.855
No restriction: *VL* = 0	5–30 km/h: *VL* = 1	1.888
Male: *S* = 0	Female: *S* = 1	0.933
Under 15 years old: *A* = 0	16 years old and above: *A* = 1	0.977
March-May: *M* = 1	June-Augst: *M* = 2	0.991
Weekday: *WD* = 0	Weekend: *WD* = 1	1.563
Wet/slippery lane: *VC* = 0	Dry lane: *VC* = 1	0.946
No alcohol involved: *VA* = 0	Alcohol involved: *VA* = 1	3.236
Lighted: *L* = 0	Not Lighted: *L* = 1	1.306
Sunny: *WT* = 0	Rainy/Snow: *WT* = 1	0.839

**Fig 4:**
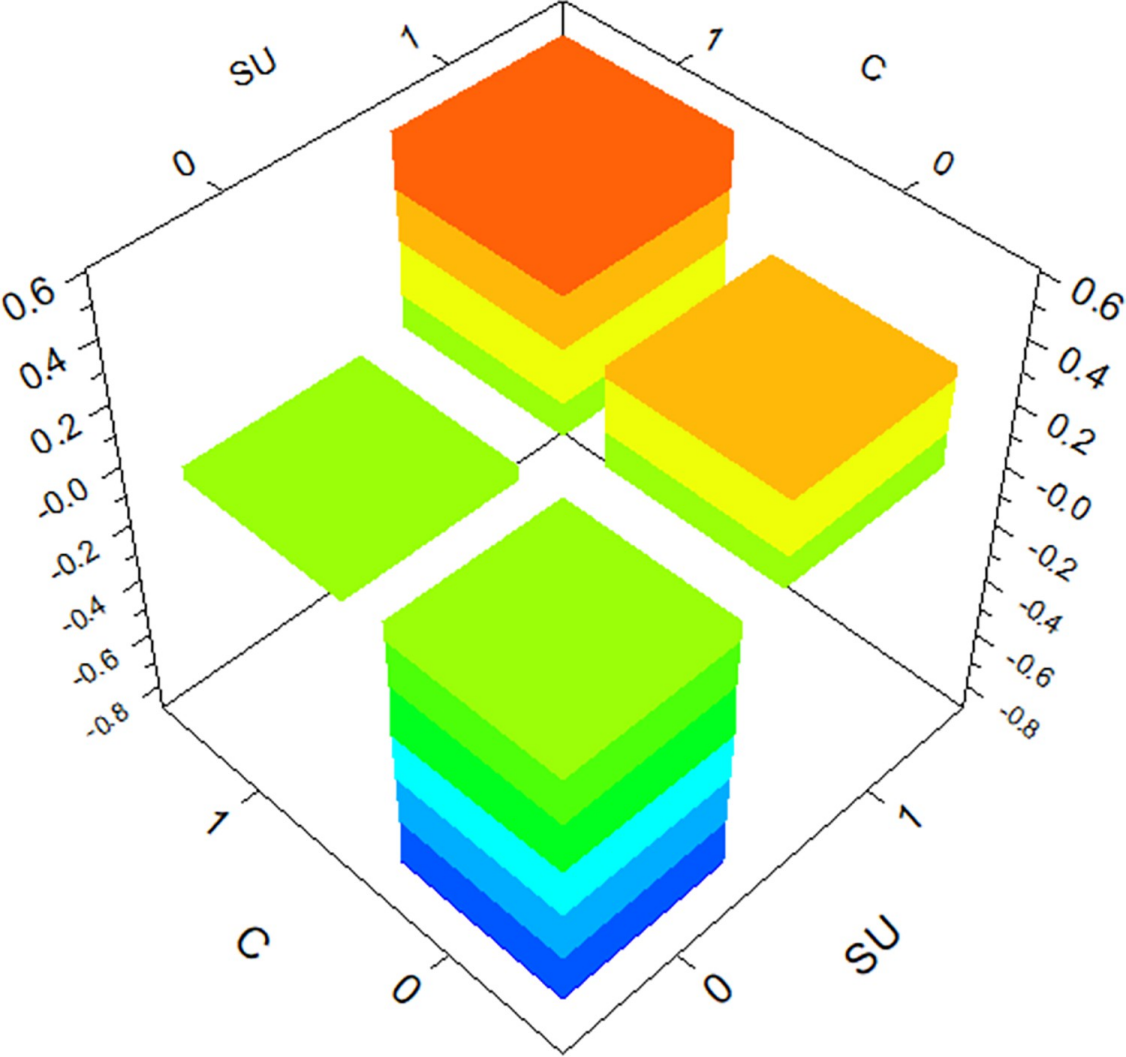
The interaction effect of *SU* and *C* on hit-and-run crashes.

**Fig 5:**
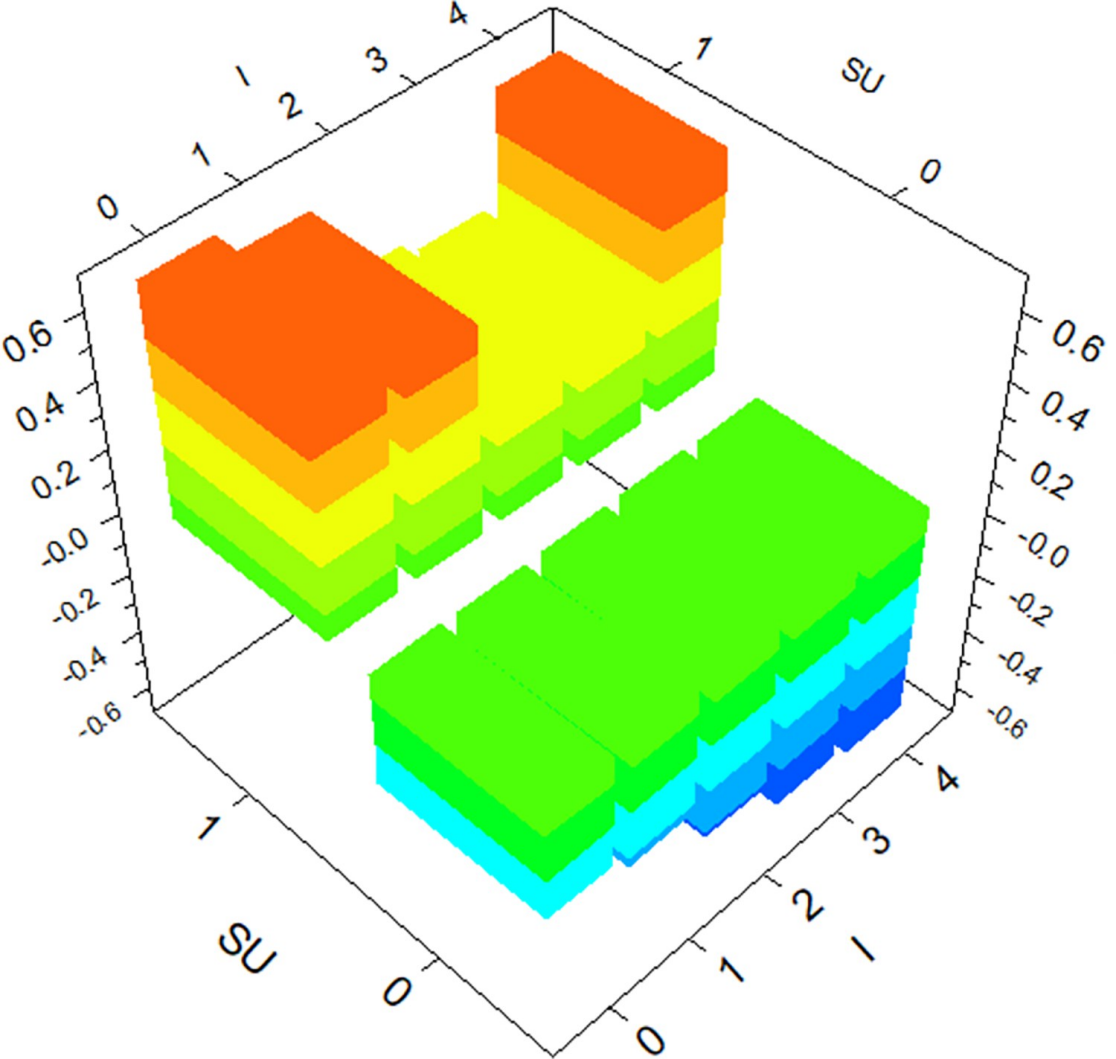
The interaction effect of *SU* and *I* on hit-and-run crashes.

**Fig 6:**
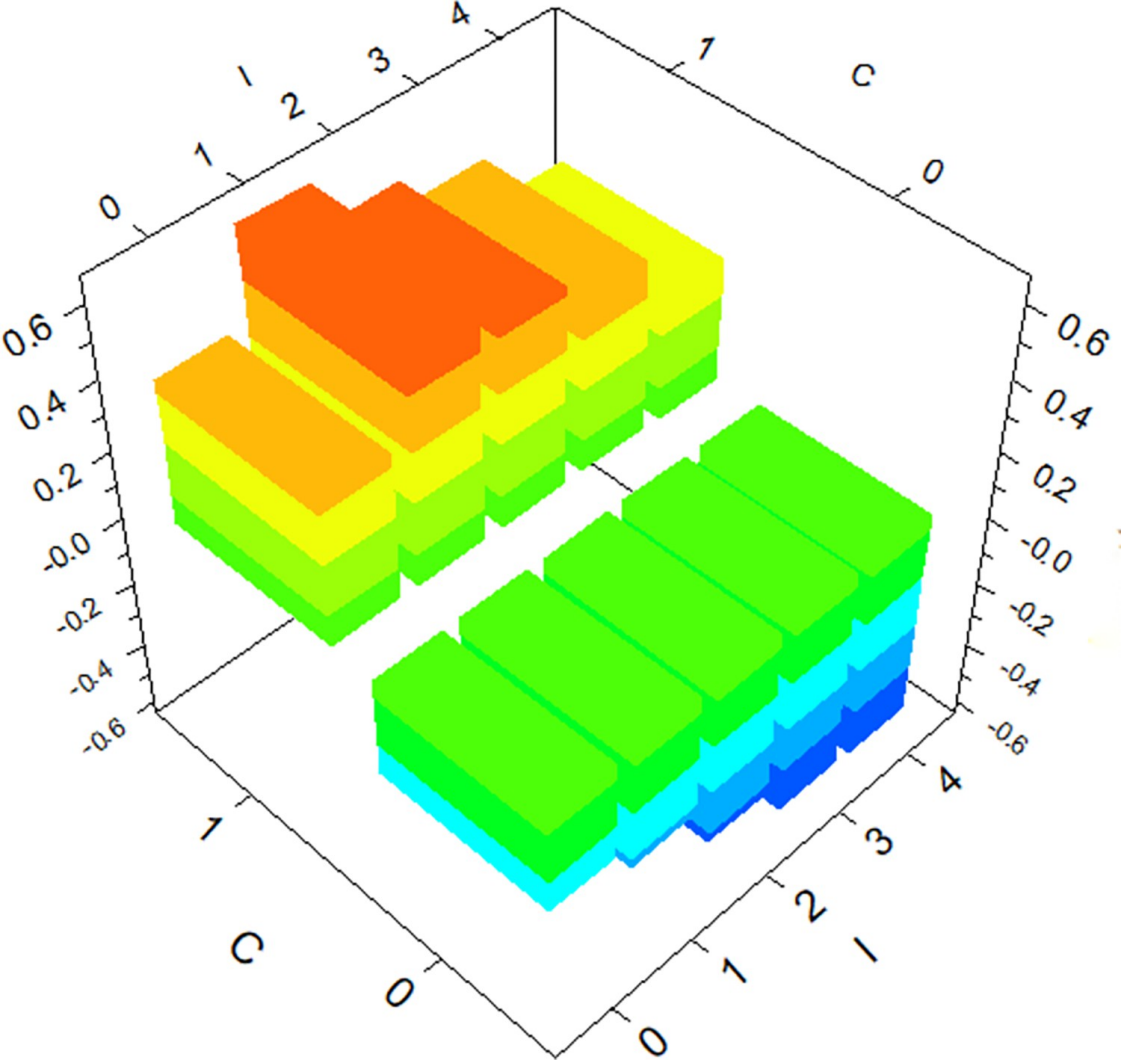
The interaction effect of *C* and *I* on hit-and-run crashes.

The GBDT model provides the degree of influence of individual variables on hit-and-run crashes and reflects the interactions between variables. The top three variables in terms of importance (*SU*, *C*, *I*) are selected for three-dimensional plots, as shown in Figs 4–6.

Under the interaction between *Cargo Body Type* (*C*) and *Special Use* (*SU*), the likelihood of hit-and-run crashes is higher for trucks used for public commercial purposes, aligning with the conclusion from the analysis of individual factors. Similarly, under the interaction between *Special Use* (*SU*) and severity of driver *Injury Severity* (*I*), the likelihood of hit-and-run crashes is higher for public commercial vehicles when drivers are not injured. Interestingly, the likelihood of hit-and-run crashes is also significant when drivers suffer fatal injuries, a factor not evident in the analysis of individual factors. Under the interaction between *Cargo Body Type* (*C*) and *Injury Severity* (*I*), the likelihood of hit-and-run crashes is higher for trucks when drivers suffer minor injuries, another aspect not apparent in the analysis of individual factors. This confirms that the occurrence of hit-and-run crashes is influenced by the interaction of multiple factors.

To assess the predictive accuracy of the model, this study compares it with Classification and Regression Trees (CART), Random Forest (RF), and Logistic Regression (LR) models using the same dataset, as shown in Table 6.

**Table 6:** Model validation comparison.

Model	NLL	Misclass	LIFT	AUC
CART	0.299	0.108	3.687	0.759
GBDT	0.282	0.096	4.087	0.803
RF	1.124	0.255	3.980	0.767
LR	0.294	0.102	3.805	0.756

To assess the fitting performance of the four models, we used negative average log-likelihood (NLL) as a measure, where a lower NLL value indicates better model fit. Misclassification rates were used to evaluate the accuracy of the models, with smaller Misclass values representing higher accuracy. Model performance improvement (LIFT) was employed to examine computational efficiency, where larger LIFT values suggest better efficiency. Additionally, the area under the curve (AUC) of the receiver operating characteristic (ROC) curves was considered to illustrate the classification capabilities of the models, with higher AUC values indicating better classification performance.

Table 6 presents a detailed comparison of the four models: CART, GBDT, RF, and LR. In terms of NLL, which measures model fit, the GBDT model achieves the best performance with an NLL of 0.282, followed closely by the LR (0.294) and CART (0.299) models. The RF model, however, performs significantly worse in this metric, with an NLL of 1.124. When evaluating misclassification rates, which assess model accuracy, the GBDT model again demonstrates superior performance with the lowest misclassification rate of 0.096. This is followed by LR (0.102) and CART (0.108), while RF lags behind with a misclassification rate of 0.255. Notably, the GBDT model achieves the highest accuracy, as indicated by the lowest misclassification rate of 0.096, outperforming the other models. In terms of fitting performance, all models performed adequately, but the GBDT model’s superior accuracy makes it stand out.

In addition to accuracy, the GBDT model shows a significant computational advantage, with a LIFT value of 4.087—far exceeding the benchmark value of 1, indicating a substantial improvement in computational efficiency. This result highlights GBDT’s ability to handle large-scale data more effectively than the other models, which is particularly important for real-time or large-volume applications such as hit-and-run crash prediction.

Moreover, GBDT also demonstrated the strongest classification capability, as evidenced by its high AUC value, making it the most reliable model for distinguishing between hit-and-run and non-hit-and-run crashes. The combination of high accuracy, computational efficiency, and classification performance confirms that GBDT is not only a robust predictive tool but also highly suitable for engineering applications, such as hit-and-run accident prevention, resource allocation, and safety analysis. These attributes make GBDT particularly advantageous in scenarios where both precision and processing speed are critical.

## Discussion

This study examines the factors influencing hit-and-run crashes through the classification of variables into five key categories: Accident Characteristics, Driver Characteristics, Vehicle Characteristics, Road Characteristics, and Environmental Characteristics. Using the Gradient Boosting Decision Tree (GBDT) model trained on 54,187 crash records, the results demonstrate strong predictive performance, with an AUC of 0.803. These findings align with, and extend, previous research on the determinants of hit-and-run behavior.

### Accident characteristics

Consistent with prior studies, such as Tay et al. [9], our findings indicate that hit-and-run behavior is more likely during crashes on weekends compared to weekdays (OR = 1.563), likely due to reduced traffic enforcement during these periods. Additionally, the likelihood of hit-and-run crashes is significantly higher during nighttime (OR = 3.855), which may be due to the perception of hit-and-run drivers that the anonymity of darkness reduces the likelihood of being detected. This result is also supported by Tay et al. [4]. The likelihood of hit-and-run incidents is lower during the summer months (June to August) (OR = 0.991), potentially due to the reduced duration of nighttime darkness, which increases the likelihood of hit-and-run drivers being detected. Furthermore, lateral crashes are more prone to hit-and-run behavior compared to frontal or rear-end crashes (OR = 1.225), a finding consistent with Jiang et al. [19]. This may be due to the difficulty of capturing lateral crashes in the dashcam footage of the victim, thereby increasing the perceived risk for hit-and-run drivers.

### Driver characteristics

The GBDT model confirms prior research on the role of driver-related factors in hit-and-run behavior. Licensed drivers are less likely to flee the scene compared to unlicensed drivers (OR = 0.977), a result consistent with studies by Macleod et al. [13]. Female drivers are less prone to hit-and-run behavior compared to males (OR = 0.933), reaffirming findings by Zhu et al. [21], who suggested that male drivers may engage in riskier behaviors. The influence of alcohol is particularly significant, with intoxicated drivers being far more likely to flee (OR = 3.236), a result that corroborates studies such as Macleod et al. [13], who attributed this behavior to the anticipation of harsher legal penalties. Interestingly, injured drivers are less likely to flee (OR = 0.951), which contrasts with the findings of Zhang et al. [2], suggesting that injury severity could be a key moderating variable in understanding hit-and-run incidents.

### Vehicle characteristics

Vehicle type emerges as one of the most influential factors in the model, with passenger vehicle drivers being significantly less likely to commit hit-and-run compared to those driving trucks (OR = 0.022). This finding expands upon earlier studies, such as those by Tay et al. [3], which did not emphasize the extent of the impact of vehicle type. The greater likelihood of fleeing in larger commercial vehicles could be attributed to the perception of greater damage or fear of higher legal repercussions. Furthermore, private vehicles are associated with a lower likelihood of hit-and-run behavior compared to public or commercial vehicles (OR = 0.004), a finding consistent with the analysis of commercial vehicle involvement in crashes by Lopez et al. [22]. The commercial stakes involved may amplify the motivation to flee.

### Road characteristics

The results also highlight the importance of road conditions. Drivers are more likely to flee on roads with lower speed limits (OR = 1.888), as lower-speed crashes may be perceived by hit-and-run drivers as less severe, thereby encouraging them to flee. This aligns with the findings of Tay et al. [4]. Hit-and-run incidents are less frequent on dry road surfaces (OR = 0.946), which corroborates the findings of Aidoo et al. [16] that drivers may feel that slippery surfaces provide them with a possible justification for the crash, making them less inclined to flee. Additionally, crashes occurring on-road, where surveillance is likely higher, are less prone to hit-and-run behavior (OR = 0.073), a result that aligns with the observation by Tay et al. [3] that better lighting and camera surveillance reduce hit-and-run incidents.

### Environmental characteristics

In terms of environmental factors, the GBDT model shows that hit-and-run crashes are less likely to occur during clear weather conditions than in rainy or snowy conditions (OR = 0.839), a finding supported by Aidoo et al. [16]. This result may be explained by drivers feeling that adverse weather conditions could provide an excuse, potentially mitigating legal consequences. Poor lighting conditions (OR = 1.306) are another significant predictor. As noted by Jiang et al. [23], darkness reduces visibility, making it easier for drivers to flee unnoticed, a trend also supported by our model’s findings.

### Key insights from the model

The GBDT model provides deeper insights compared to traditional models, such as logistic regression, by revealing the complex interactions between variables. For example, while earlier studies have indicated that vehicle type and driver alcohol consumption are influential, our model demonstrates that these factors interact in ways that amplify the likelihood of hit-and-run behavior. Specifically, the influence of vehicle type, combined with commercial use, emerges as one of the most critical determinants, a finding that has not been consistently emphasized in prior research. Additionally, the model’s ability to rank variables by importance offers a clearer understanding of how injury severity, road conditions, and environmental factors collectively influence the decision to flee the scene of a crash.

## Conclusions

This study extracts hit-and-run data from the Crash Report Sampling System (CRSS) database and develops an innovative hit-and-run classification prediction model using Gradient Boosting Decision Trees (GBDT). The novelty of this research lies in applying GBDT to capture complex interactions between variables that influence hit-and-run behavior, offering deeper insights compared to traditional models. Hit-and-run crashes represent a covert form of traffic violation influenced by various driver and environmental factors, and this study focuses on key variables such as Crash Type and Relation to Trafficway, revealing their significant roles in such incidents.

In addition to the GBDT model, CART, RF, and LR models are developed for comparison. The study leads to the following important conclusions:

1. **Impact of *relation to trafficway* and *crash type*:** These two factors rank 4th and 5th in importance, respectively, demonstrating their significant relationship to hit-and-run crashes. Understanding these factors enables targeted interventions in specific areas or situations prone to hit-and-run behavior.
2. **Variable interactions and visualizations:** The GBDT model not only quantifies the impact of individual variables but also captures interactions between them. Visualizations developed from the model provide valuable insights into how these factors work together to influence hit-and-run behavior, which can inform safety measures and policy decisions.
3. **GBDT model innovation and superiority:** The GBDT model shows superior performance compared to CART, RF, and LR in terms of computational efficiency, prediction accuracy, and classification capability. This makes it a robust tool for hit-and-run crash prediction and prevention, with clear advantages for safety analysis and engineering applications.

The novelty of this study lies in its application of the GBDT model to predict hit-and-run behavior by revealing intricate, non-linear interactions among variables, while maintaining a high level of interpretability. The model’s ability to provide clear insights into variable importance ensures that both researchers and policymakers can understand how individual factors impact hit-and-run likelihood. The practical application value of this research is substantial, as it provides policymakers and law enforcement agencies with actionable insights to develop targeted preventive strategies. The model’s ability to predict high-risk conditions, such as road types, times of day, and vehicle categories, enables more effective allocation of resources, deployment of surveillance systems, and enhanced road safety measures. Furthermore, the findings can guide the development of public awareness campaigns and training programs focused on reducing hit-and-run incidents.

While this study provides valuable insights, it also has certain limitations, primarily due to constraints in the available data:

1. **Driver psychological factors:** This study does not incorporate psychological factors such as driver stress, fatigue, or distraction, which are known to influence driving behavior. These factors cannot be considered because the CRSS database does not include psychological or cognitive data. This limitation could be addressed in future research by collecting additional data, such as driver heart rate, eye-tracking, or stress levels, to capture the psychological state of drivers at the time of the crash.
2. **Spatial and temporal limitations:** The data used in this study are geographically limited to certain regions, and only specific time frames are considered. This may reduce the model’s generalizability to other locations or time periods. To address this limitation, expanding the dataset to include data from diverse geographical regions and multiple years would improve the model’s robustness. Future studies could focus on conducting cross-regional analyses or longitudinal studies to better understand how hit-and-run behavior varies across different contexts and over time.
3. **Unmeasured environmental factors:** While this study accounts for several environmental variables, such as weather and road conditions, other factors—such as pedestrian activity and road infrastructure—are not included due to data limitations. To address this limitation, incorporating real-time traffic data from advanced sources could enhance the model’s accuracy. Future research could integrate multiple data sources to capture a more comprehensive view of the traffic environment.
